# How does teacher collaboration drive technology adoption? A PLS-SEM analysis of PISA 2022 data

**DOI:** 10.3389/fpsyg.2026.1790345

**Published:** 2026-03-16

**Authors:** Rui Liu, Fan Liu, Runlin Li, Bin Jing

**Affiliations:** 1 School of Intelligent Technology, Geely University of China, Chengdu, China; 2 School of Accounting, Chengdu College of Arts and Science, Chengdu, China; 3 Faculty of Education, Shaanxi Normal University, Xi'an, China

**Keywords:** PISA, school climate, self-efficacy, teacher collaboration, technology adoption

## Abstract

**Introduction:**

While teacher collaboration is widely recognized as a driver of educational reform, the specific mechanisms by which it translates into individual technology adoption remain underexplored. This study addresses this gap by constructing a theoretical framework that synthesizes Communities of Practice and Social Cognitive Theory, focusing on how social interactions are internalized as psychological resources.

**Methods:**

Utilizing the large-scale global dataset from the PISA 2022 assessment (*N* = 38,747), we employed Partial Least Squares Structural Equation Modeling (PLS-SEM) to test a chain mediation model. The analysis examined the interplay between teacher collaboration, school climate, and teacher self-efficacy as predictors of instructional technology adoption.

**Results:**

The results reveal a complementary partial mediation mechanism. Specifically, teacher collaboration is associated with technology adoption not only directly by facilitating resource exchange but also through three distinct indirect pathways: (1) an environmental pathway mediated by school climate; (2) a psychological pathway mediated by teacher self-efficacy; and (3) a serial pathway where school climate enhances self-efficacy. These findings delineate a consistent mechanism of socialization, internalization, and externalization, underscoring that internal psychological states are significantly linked to the perceived control over both internal and external factors.

**Discussion:**

The results validate an integrated Social-Cognitive Integration Framework, clarifying how social interactions translate into individual behavioral change. The mediation effect highlights that school climate and self-efficacy serve as the fundamental pillars promoting technology adoption. Implications suggest that university-based teacher education should prioritize collaborative competencies to build preservice teachers’ psychological resilience, while the school administrators must cultivate “psychologically safe” environments to sustain the vitality of digital transformation.

## 1 Introduction

In the wake of the global digital transformation, integrating technology into daily instruction has become a strategic imperative for educational systems worldwide (Haleem et al., 2022). Although hardware infrastructure and digital resources have seen substantial investment over the past decade (OECD, 2023), teachers’ behavioral engagement in classroom technology use remains inconsistent and faces numerous obstacles (Fraillon, 2025). Research indicates that the bottleneck for sustainable technology integration is often not technical availability, but rather the complex interplay of social and psychological factors within the school environment (Ertmer et al., 2012).

To address these barriers, Teacher Collaboration has become a crucial means for promoting educational reform and professional development (Hargreaves, 2021; Vangrieken et al., 2015). Unlike solitary professional development, collaboration offers a dynamic platform for resource exchange, reflective dialogue, and mutual support (Little, 1990; Moolenaar, 2012). However, while empirical evidence broadly supports the positive link between collaboration and technology adoption, the precise mechanisms underlying this relationship remain underexplored. Existing research often focuses on isolated pathways, exploring collaboration either from a sociological perspective to improve organizational climate (Çoban et al., 2023; Friesen and Brown, 2022; Ninković et al., 2022) or from a psychological perspective to enhance individual confidence (Li, 2024; Mather and Visone, 2024; Tatar et al., 2025). Few studies have integrated these environmental and cognitive factors into a unified mediating mechanism, leaving practitioners with limited guidance on how to convert external social interactions into concrete instructional changes.

To bridge this research gap, this study proposes an integrated framework synthesizing Communities of Practice (CoP; Wenger, 1998) and Social Cognitive Theory (SCT; Bandura, 1986). We posit that the role of teacher collaboration operates through a multi-path mechanism rather than a simple linear trajectory. Specifically, SCT provides the theoretical micro-foundation for this linkage, positing that collaborative networks function as critical sources of “vicarious experience” and “social persuasion” (Bandura, 1986). By observing peers successfully navigate digital tools, teachers reduce their perceived uncertainty and build the psychological resilience necessary for adoption. Consequently, this study examines how collaboration facilitates technology adoption directly, while simultaneously functioning through two distinct mediators: School Climate (as an environmental facilitating condition) and Teacher Self-Efficacy (as a key factor of internal cognition). Furthermore, we explore a serial mediation model to elucidate how supportive organizational environments are progressively internalized into individual beliefs and subsequently associated with instructional behaviors.

Drawing on the large-scale global dataset from the Programme for International Student Assessment (PISA) 2022, this study employs Partial Least Squares Structural Equation Modeling (PLS-SEM; Hair et al., 2019) to empirically examine the hypothesized mediation framework. By deconstructing specific parallel and serial pathways, this research aims to provide school leaders and policymakers with actionable evidence for fostering a holistic ecosystem that empowers teachers to navigate and sustain digital transformation.

## 2 Literature review

### 2.1 Theoretical framework

To elucidate the underlying mechanisms by which teacher collaboration drives technology adoption, this study proposes an integrated theoretical framework synthesizing SCT and CoP. While SCT offers a robust foundation for understanding the interplay between environmental factors and individual cognition in shaping behavior, it offers limited insight into the genesis of organizational contexts, such as school climate. The incorporation of CoP theory addresses this limitation by explaining how social interactions among teachers cultivate a supportive organizational environment.

#### 2.1.1 Social cognitive theory: the environment-cognition-behavior interplay

Drawing upon SCT, this study adopts the framework of Triadic Reciprocal Determinism to examine the dynamic interaction among environmental, personal, and behavioral factors (Bandura, 1986). In the proposed model, these components are operationalized as school climate (teachers’ perceptions of organizational support and interpersonal trust), teacher self-efficacy (beliefs in managing digital classrooms), and technology adoption, respectively. SCT posits that environmental factors not only influence behavior directly but also act as catalysts for behavioral change by being internalized into cognitive beliefs (Bandura, 2001). Specifically, a supportive school climate provides essential sources of social persuasion and vicarious experiences, which bolster teachers’ self-efficacy and ultimately facilitate the sustained integration of technology in instruction (Liu et al., 2025).

#### 2.1.2 Communities of practice: the social construction of the environment

While SCT emphasizes the influence of the environment, it often treats external conditions as a given, offering limited insight into their socio-genesis. To elucidate the origins of a “supportive school climate,” this study incorporates CoP theory (Wenger, 1998). CoP theory posits that learning and socialization occur within the context of shared participatory practices. According to Wenger, when members engage in frequent mutual engagement and joint enterprise centered on a shared vision, they transcend mere information exchange to construct a shared identity and collective social capital (Lave and Wenger, 1991). In this study, teacher collaboration serves as the concrete manifestation of such engagement. Through collaboration practice, teachers dismantle professional isolation and establish interpersonal networks grounded in reciprocity; at the organizational level, these networks manifest as a positive and cohesive school climate (Vangrieken et al., 2015).

#### 2.1.3 Rationale for theoretical integration

Collaboration has long been established as an effective mechanism for navigating shifts in educational environments and achieving reform objectives (Datnow, 2020; Egodawatte et al., 2011; Nguyen and Ng, 2020). As the locus of educational reform shifts from mere content to the instructional context, collaboration has emerged as an imperative for the effective restructuring of curriculum and pedagogical practices (Datnow, 2020; Manouchehri, 1997). This collaborative necessity is evident across diverse cultural contexts. For instance, in Finland, the integration of collaborative elements is regarded as pivotal for fostering purposeful pedagogical practices (Tirri, 2014), and in Myanmar, inter-institutional collaboration is essential for ensuring the sustainable implementation of educational reforms (Sin, 2021). Empirical evidence further demonstrates that collaborative mechanisms translate directly into enhanced instructional capacity. For example, collaboration within Professional Learning Communities facilitates the cross-fertilization of ideas, driving substantive changes in pedagogical practices (Ryan et al., 2009). Furthermore, through co-planning and shared goal-setting, teachers not only deepen their subject matter knowledge but also directly bolster their technological skills (Egodawatte et al., 2011). Additionally, shared responsibility is identified as a critical mechanism for disseminating innovative practices amidst school transformation (Nguyen and Ng, 2020).

However, the efficacy of collaboration is not automatic; rather, it is deeply embedded within social structures and the quality of interactions. From the perspective of Social Network Theory (SNT), “social embeddedness” within schools can act as either a catalyst or a barrier to reform, depending on the specific social configurations underpinning collaborative efforts (Moolenaar, 2012). For collaboration to yield meaningful outcomes, high-quality dialogue must serve as a linchpin for developing pedagogical expertise, while teachers navigate complex social dynamics involving agency, power, and social justice (Datnow, 2020; Horn and Kane, 2015). Despite these potential benefits, collaboration also entails inevitable drawbacks such as conflict, increased workload, and a potential loss of autonomy (Weddle, 2022), requiring a deeper unpacking of “practice” within complex learning experiences (Lampert, 2010).

Synthesizing the preceding analysis, a critical gap emerges in the extant literature: while empirical evidence confirms that collaboration facilitates skill enhancement under optimal conditions, its efficacy is inevitably constrained by complex social networks. Current scholarship has yet to fully elucidate the specific psychological and environmental mechanisms by which socially embedded collaboration surmounts these social dynamics to ultimately translate into sustained technology adoption. To bridge this gap, this study proposes an integrative explanatory framework synthesizing CoP and SCT. According to Bandura’s (1986) Social Cognitive Theory, individual behavior change is deeply rooted in social observation. In the context of this study, teacher collaboration serves as a primary mechanism for “vicarious learning.” Unlike isolated experimentation, collaboration allows teachers to observe the successful technology practices of their peers. This observational process generates “vicarious efficacy”—the belief that “if they can do it, I can do it too”—which directly counteracts the anxiety associated with new technology (Bandura, 1997). Therefore, SCT provides the theoretical bridge explaining why structural collaboration translates into individual behavioral adoption. Based on this, we posit a sequential mechanism wherein teacher collaboration fosters a supportive school climate, which is subsequently internalized into teacher self-efficacy, thereby catalyzing technology adoption. Based on that, we posit a sequential mechanism wherein teacher collaboration fosters a supportive school climate, which is subsequently internalized into teacher self-efficacy, thereby catalyzing technology adoption.

### 2.2 Teacher collaboration and its psychological impacts

School climate reflects the collective perceptions and experiences of school members regarding their organizational environment, representing a social relational network anchored in shared norms, goals, and interpersonal interactions rather than mere physical attributes (Cohen et al., 2009). From this perspective, school climate is conceptualized as a form of organizational social capital, manifesting primarily through teacher-student relationships and collegial trust (Hoy and Sweetland, 2001; Liu et al., 2025). However, traditional bureaucratic school structures are often characterized by professional isolation, which severs the flow of information and emotional support, thereby imposing significant constraints on teachers (Ostovar-Nameghi and Sheikhahmadi, 2016). This structural isolation ultimately erodes the foundation of organizational trust and negatively impacts teacher job satisfaction, necessitating a shift toward more collaborative professional environments (Tabancalı, 2016).

Drawing on CoP and SNT, the organizational environment is conceptualized not as a static physical entity but as one deeply embedded within complex networks of social interaction. This “social embeddedness” dictates the flow of resources and the structure of relational trust, with recent empirical evidence identifying teacher collaboration as a pivotal mechanism for reconfiguring these social structures to foster a positive climate (Moolenaar, 2012). Teacher collaboration is a multifaceted construct that extends beyond mere social interaction to include specific dimensions such as instructional planning, peer observation, and professional dialogue. For instance, Tripon (2022) emphasized that supporting teachers through such collaborative frameworks is essential for promoting complex instructional skills, such as computational thinking in STEM education, thereby suggesting that deep professional interaction is a prerequisite for effective technology integration. Specifically, collaboration cultivates “shared responsibility” by enabling teachers to move beyond the exchange of technical know-how to collectively distribute the burden of instructional improvement through practices such as co-teaching and action research (Nguyen and Ng, 2020). These profound interactions transform fragmented individual tasks into a collective professional commitment, significantly enhancing organizational cohesion. Moreover, collaboration serves to mitigate the social tensions and power dynamics inherent in educational reforms, fostering a psychologically safe environment (Datnow, 2020). By establishing cross-hierarchical partnerships, collaboration empowers teachers with the agency to collectively navigate the anxiety and uncertainty associated with change, thereby alleviating professional strains and encouraging experimentation with new instructional practices (Sin, 2021).

Beyond shaping the organizational climate, teacher collaboration serves as a primary source of psychological empowerment. According to Bandura’s (1997) SCT, vicarious experience acts as a critical driver of self-efficacy; by observing peers successfully integrating technology or collectively resolving technical hurdles, teachers gain substantive evidence of their own latent capabilities. This modeling effect facilitates a direct cognitive appraisal of their proficiency, allowing them to bolster their confidence in mastering digital tools independently of broader organizational shifts (Horn and Kane, 2015). Consequently, collaborative interactions function not only as a social support system but also as a catalytic cognitive resource that directly elevates individual instructional self-efficacy.

### 2.3 The role of school climate in digital transformation

Teacher self-efficacy refers to a teacher’s belief in their ability to organize and execute the actions required for specific teaching tasks (Bandura, 1997), serving as a primary driver of task performance, goal achievement, and work efficiency (Li, 2023; Sun and Yoon, 2025; Yan et al., 2025). In the context of digital transformation, technology integration is often fraught with high levels of uncertainty and pedagogical risks, such as technical malfunctions and the potential loss of classroom management (Howard, 2013; Howard and Gigliotti, 2016). Such environmental ambiguity presents a formidable challenge to teachers’ confidence, rendering their psychological state highly contingent upon external support (Hülshoff et al., 2025; Khlaif et al., 2023).

According to SCT, environmental factors constitute a critical antecedent to the development of self-efficacy. Moving beyond the primacy of individual mastery experience, Moolenaar (2012) argues from a social network perspective that teachers are “socially embedded” agents whose professional beliefs are fundamentally reshaped by the school structure. This social embeddedness implies that a school climate characterized by organizational trust and shared norms bolsters teacher self-efficacy through two distinct mechanisms. First, a positive climate serves as a rich reservoir of vicarious experience; within an organizational milieu of mutual trust, teachers are afforded amplified opportunities for high-quality dialogue and peer observation, which facilitate the acquisition of instructional expertise (Horn and Kane, 2015; Reilly, 2017). Witnessing colleagues successfully integrate technology within similar contexts creates a “modeling effect” that transmits signals of competence, thereby strengthening the observer’s conviction in their own capabilities (Mather and Visone, 2024). Second, a supportive climate fosters psychological safety, effectively mitigating the trepidation and anxiety often associated with complex educational reforms (Henderson and Corry, 2021). In an atmosphere of mutual trust, errors are construed as learning opportunities rather than grounds for punitive measures, thereby alleviating the tensions inherent in pedagogical experimentation (Datnow, 2020; Sin, 2021). By perceiving sustained emotional support from peers, teachers’ fear of technological risk is significantly diminished, empowering them to engage more confidently in digital instruction (Dong et al., 2020; Howard and Gigliotti, 2016; Khlaif et al., 2023).

Conversely, in environments characterized by a deficit of trust or acute professional isolation, fragmented social networks impede the essential flow of knowledge and emotional support. Such isolation compels teachers to perceive technological experimentation as a high-risk endeavor, thereby stifling the development of self-efficacy. Beyond its role as a psychological incubator, a positive school climate functions as a direct environmental facilitator for behavioral change (Maral, 2025). In schools defined by mutual trust, established “social norms” actively encourage innovation and foster a tolerance for failure. This supportive atmosphere reduces the perceived risks of technology adoption, effectively serving as a “facilitating condition” that lowers the threshold for implementation (Venkatesh et al., 2003). Consequently, independent of their internal efficacy beliefs, teachers are more likely to adopt technology when the organizational environment renders the process structurally easier and psychologically safer.

### 2.4 Teacher self-efficacy and technology adoption

In this study, technology adoption encompasses not only the frequency of digital tool usage but also the depth and breadth of its integration into pedagogical practices. Within the fields of information systems and educational technology, teacher self-efficacy is widely acknowledged as the most robust psychological predictor of technology-related behavioral intention (Compeau and Higgins, 1995). According to SCT, self-efficacy extends beyond a static assessment of competence, serving instead as a fundamental source of instructional agency amidst digital transformation (Gordon et al., 2023; Min, 2023). This sense of agency predisposes highly efficacious teachers to perceive complex technical tasks, such as blended learning design, as challenges to be mastered rather than threats to be avoided. Consequently, they exhibit a greater propensity to proactively explore innovative tools to resolve pedagogical dilemmas, moving beyond passive compliance with administrative mandates toward active professional engagement (Datnow, 2020).

Furthermore, self-efficacy enhances behavioral persistence in disseminating and sustaining innovative practices (Gordon et al., 2023), a quality indispensable for navigating the technical disruptions and instructional setbacks that are frequent in the early stages of technology integration (Alenezi, 2017; Karasavvidis and Kollias, 2017; Nguyen and Ng, 2020). Empirical evidence suggests that when confronting such obstacles, teachers who are confident in their digital classroom management exhibit superior resilience. Rather than succumbing to temporary failures or adopting “avoidance strategies” driven by a fear of failure, these efficacious practitioners sustain their efforts until technology is successfully internalized into their daily pedagogical routines (Hatlevik, 2017). Conversely, teachers with low self-efficacy often cope with insecurity by reducing the scope of teaching and avoiding complex tasks, confining technology application to the surface level within their “comfort zone” (Nordlöf et al., 2019).

### 2.5 Research hypotheses

**Figure 1:**
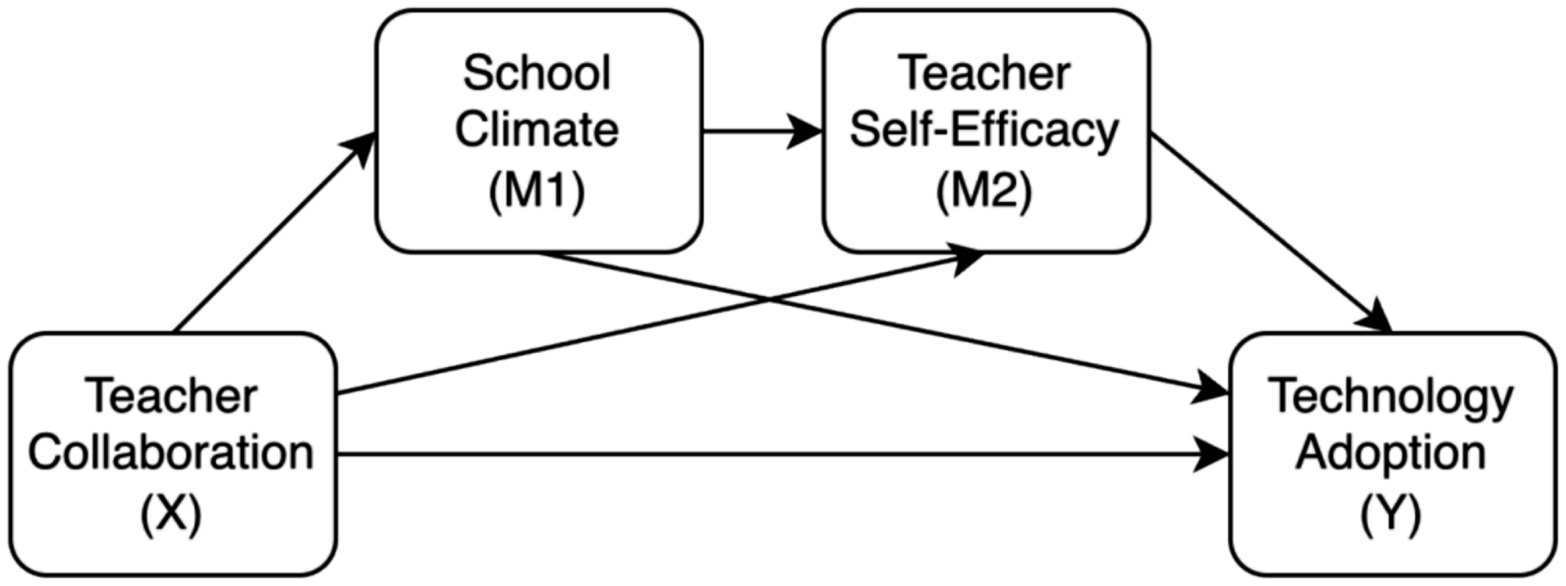
The hypothesized mediation model.

Synthesizing the theoretical underpinnings of SCT and CoP, this study postulates a comprehensive mediating framework (see Figure 1). While prior scholarship has predominantly scrutinized isolated pathways, such as the mediating role of school climate (Liu et al., 2025), it has yet to fully explicate the sequential process by which environmental resources are converted into individual psychological capital. The present study argues that the impact of teacher collaboration on technology adoption is realized through three distinct trajectories. First, collaboration acts as a mechanism of social construction that optimizes the school climate; this climate, in turn, serves as a critical facilitating condition that directly supports technology adoption by reducing perceived risks. Second, collaborative interactions serve as a direct source of psychological empowerment, providing vicarious experiences that bolster self-efficacy and propel adoption. Accordingly, we propose:

*H1*: School climate mediates the relationship between teacher collaboration and technology adoption.

*H2*: Teacher self-efficacy mediates the relationship between teacher collaboration and technology adoption.

Beyond these parallel effects, we posit a serial mediation mechanism that elucidates the transformative process from external interaction to internal belief. Specifically, collaboration initially fosters a supportive school climate, which subsequently functions as a nurturing reservoir of social and emotional resources that enhances teacher self-efficacy. This heightened efficacy then serves as the final psychological driver of sustained technology adoption. This sequential pathway captures how teacher collaboration, as a collective practice, is gradually internalized into personal agency before being manifested as innovative instructional behavior. Consequently, we propose the following serial hypothesis:

*H3*: Teacher collaboration exerts a significant indirect influence on technology adoption through the serial mediating effects of school climate and teacher self-efficacy.

## 3 Methodology

### 3.1 Data source and participants

Data for this study were drawn from the global dataset of Programme for PISA 2022, conducted by the Organisation for Economic Co-operation and Development (OECD). PISA employs a two-stage stratified sampling design: schools are first sampled within participating countries or economies, followed by a random selection of students and teachers within those schools. This rigorous methodology ensures a high degree of sample representativeness (OECD, 2023). Although PISA primarily focuses on student academic performance, its accompanying Teacher Questionnaire provides rich empirical data for investigating instructional practices, school organizational environments, and digital transformation.

This study specifically utilized the dataset from the Teacher Questionnaire. To ensure the robustness of the analysis, samples containing missing values on core variables (e.g., teacher collaboration, technology adoption) were excluded during the data preprocessing phase. Consequently, the final sample comprises 38,747 teachers from all participating countries and economies available in the PISA 2022 teacher questionnaire dataset. The sample spans a diverse group of teachers across varying genders, years of teaching experience, and school backgrounds, demonstrating broad representativeness.

### 3.2 Measures

Data were derived from the OECD PISA 2022 Teacher Questionnaire. To ensure transparency and replicability regarding construct operationalization, the specific PISA item codes, question wording, and sources for all latent variables are systematically detailed in Table 1.

**Table 1:** Measurement items and construct operationalization.

Construct/Latent variable	PISA item code	Question description (item wording)
Teacher collaboration (X)	TC046Q04NA	Exchange teaching materials with colleagues.
	TC046Q05NA	Engage in discussions about the learning development of specific students.
	TC046Q06NA	Work with other teachers in my school to ensure common standards in evaluations for assessing student progress.
	TC046Q07NA	Attend team conferences.
School climate (M1)	TC241Q01JA	Teachers can rely on the school management team for professional support.
	TC241Q02JA	The principal has confidence in the expertise of the teachers.
	TC241Q03JA	Even in difficult situations, my colleagues know they can trust me.
	TC241Q04JA	Teachers can rely on each other.
Teacher self-efficacy (M2)	SEFFCM (Derived)	Index of Classroom Management Self-Efficacy, derived from teachers’ responses to: “To what extent can you do the following?” Calm a student who is disruptive. Control disruptive behavior in the classroom. Get students to follow classroom rules. Make my expectations about student behavior clear.
Technology adoption (Y)	TC169Q01HA	Tutorial software or practice programs.
	TC169Q02HA	Digital learning games.
	TC169Q03HA	Word-processors or presentation software (e.g., Microsoft Word, PowerPoint).
	TC169Q05HA	Multimedia production tools (e.g., media capture and editing, web production).
	TC169Q08HA	Simulations and modelling software.
Control variable	TC007Q01NA	Number of years working as a teacher in total.

Given that the indicators for each variable are conceptualized as manifestations of the underlying latent traits, all constructs were modeled as reflective measurement models (Hair et al., 2019). The specific measures are described below:

Teacher Collaboration (Independent Variable). This construct measures the frequency and depth of teachers’ professional interactions. It captures key dimensions of collaborative practice, including instructional coordination (e.g., exchanging materials) and collaborative problem-solving (e.g., engaging in discussions about student development). We utilized four items from the TC046 series (e.g., “Exchange teaching materials with colleagues,” “Co-engage in lesson planning”). Items were rated on a 6-point scale, ranging from “Never” (1) to “Once a week or more” (6). In the present sample, the Cronbach’s *α* coefficient for this scale was 0.795, indicating satisfactory internal consistency.

School Climate (Mediator). School climate is defined as the degree of organizational trust and support perceived by teachers. Measurement involved four items from the TC241 series (e.g., “Teachers can rely on the school management team for support,” “Teachers can rely on each other”). Responses were recorded on a 4-point scale anchored at “Strongly disagree” (1) and “Strongly agree” (4). The Cronbach’s *α* coefficient was 0.815.

Teacher Self-Efficacy (Mediator). This study focuses on efficacy in classroom management, utilizing the derived variable SEFFCM (Classroom management self-efficacy) provided by PISA. This index is based on teachers’ self-assessments of their capability to maintain classroom order (e.g., “To what extent can you calm a student who is disruptive?”). Classroom management efficacy was selected as a critical proxy for agentic capability, as maintaining instructional order is a foundational prerequisite for implementing technology-enhanced lessons. The variable represents Weighted Likelihood Estimates generated via Item Response Theory. As an official PISA composite index, its validity and reliability have been rigorously verified in the OECD technical report (OECD, 2023).

Technology adoption (dependent variable). Technology adoption denotes the frequency with which teachers employ various digital tools in classroom instruction. Five representative items were selected from the TC169 series, encompassing a spectrum from basic tools (e.g., “word-processing software”) to advanced applications (e.g., “simulation software, digital learning games”). This measure distinguishes active pedagogical use from mere access. Items were measured on a 5-point scale ranging from “Never or almost never” (1) to “Every day or almost every day” (5). The Cronbach’s *α* coefficient was 0.772.

Control variables. To isolate the potential confounding effects of individual career stages on the results, Teacher Experience (TC007) was incorporated into the model as a control variable. It was measured using a single item reflecting the number of years the participant has worked as a teacher.

### 3.3 Data analysis strategy

Given the specific characteristics of the data and the research objectives, PLS-SEM was employed for hypothesis testing. The data analysis was performed using Python (Version 3.10). Specifically, the PLS-SEM algorithm and mediation analyses were implemented using custom scripts. Data management and preprocessing were handled using pandas and pyreadstat. The core statistical modeling relied on scikit-learn for principal component analysis and linear regression, and statsmodels for multicollinearity diagnostics. Furthermore, the bootstrapping procedure (2,000 subsamples) was executed using the resample module from scikit-learn to ensure robust significance testing. Although the sample size is large and the descriptive statistics (Table 2) indicate a relatively normal distribution, PLS-SEM was selected over Covariance-Based SEM (CB-SEM) for two primary reasons. First, the data are derived from Likert scales, which are inherently ordinal in nature. PLS-SEM is a non-parametric method that does not impose the strict distributional assumptions required by CB-SEM and is particularly robust for handling ordinal data structures (Hair et al., 2019). Second, the primary objective of this study is identifying the key driver constructs and maximizing the explained variance ( *R* ^2^) of the target variable (Technology Adoption), rather than strictly confirming a theoretical model fit, making PLS-SEM the more appropriate methodological choice (Hair et al., 2021).

**Table 2:** Descriptive statistics of latent constructs.

Construct	Mean	SD	Skewness	Kurtosis
Teacher collaboration (X)	4.399	1.163	−0.479	−0.574
School climate (M1)	3.263	0.533	−0.394	0.331
Teacher self-efficacy (M2)	0.559	1.034	−0.751	0.091
Technology adoption (Y)	2.099	0.654	0.599	0.06
Teacher experience (Control)	16.446	9.93	0.503	−0.364

The analysis followed the two-stage evaluation procedure recommended by Hair et al. (2021). In the first stage, we assessed the reliability and validity of the reflective measurement model. Reliability was evaluated using Cronbach’s ⍺ and Composite Reliability (CR), with the expectation that both indices exceed the threshold of 0.70. Convergent validity was examined through item outer loadings and Average Variance Extracted (AVE); specifically, outer loadings were required to be significant and greater than 0.708, while AVE values needed to surpass 0.50 (Hair et al., 2019). Furthermore, discriminant validity was assessed via the Fornell-Larcker criterion, ensuring that the square root of the AVE for each latent variable exceeded its correlations with other constructs (Fornell and Larcker, 1981). In the second stage, upon confirming the adequacy of the measurement model, we proceeded to evaluate the structural model. First, Variance Inflation Factors (VIF) were calculated to rule out multicollinearity among predictor variables, using a threshold of 3.0 (Hair et al., 2019). The model’s explanatory power was gauged using the coefficient of determination (*R*^2^). To test the proposed hypotheses, we employed a bootstrapping procedure configured with 2,000 subsamples to compute T-statistics and significance levels for the path coefficients (Chin, 1998). Finally, to test the proposed multiple mediation hypotheses, we examined the significance of both specific indirect effects (for H1 and H2) and the serial mediation effect (for H3). This was achieved by calculating the bias-corrected 95% confidence intervals (CI) for each specific indirect path, following the approach suggested by Preacher and Hayes (2008).

## 4 Results

### 4.1 Descriptive statistics

The Table 2 presents the means, standard deviations, skewness, and kurtosis values. As shown in the table, the skewness and kurtosis indices for all constructs ranged between −1 and +1, suggesting that the data distribution is relatively normal. Although PLS-SEM is often chosen for its ability to handle non-normal data, it performs equally well with normal data. More importantly, given the study’s objective to maximize the explained variance ( *R* ^2^) of the dependent variable rather than assessing global model fit, PLS-SEM remains the most appropriate analytical approach (Hair et al., 2019).

### 4.2 Measurement model assessment

First, we assessed the internal consistency reliability and convergent validity of the latent variables. As detailed in Table 3, Cronbach’s ⍺ coefficients for all multi-item constructs ranged from 0.772 to 0.815, while CR values spanned from 0.846 to 0.878. All indicators surpassed the recommended threshold of 0.70 (Hair et al., 2019), signifying the robust reliability of the measurement scales.

**Table 3:** Measurement model evaluation.

Construct	Alpha	CR	AVE	Result
X_Collab	0.795	0.867	0.621	Pass
M1_Climate	0.815	0.878	0.643	Pass
Y_Adopt	0.772	0.846	0.525	Pass
M2_Efficacy	1.000	1.000	1.000	Pass
Ctrl_Exp	1.000	1.000	1.000	Pass

Regarding convergent validity, the AVE values for all latent variables ranged from 0.525 to 0.643. These figures exceeded the benchmark of 0.50, indicating that the constructs accounted for more than 50% of the variance in their respective indicators.

Subsequently, discriminant validity was evaluated using the Fornell-Larcker criterion. As presented in Table 4, the bolded values on the diagonal represent the square roots of the AVEs (ranging from 0.724 to 1.000). Crucially, these values were consistently higher than the off-diagonal elements in their corresponding rows and columns, which represent the correlations between constructs. This pattern demonstrates that the constructs are statistically distinct, thereby confirming that the measurement model possesses satisfactory discriminant validity (Fornell and Larcker, 1981).

**Table 4:** Discriminant validity (Fornell-Larcker).

Construct	X_Collab	M1_Climate	Y_Adopt	M2_Efficacy	Ctrl_Exp
X_Collab	0.788	0.251	0.301	0.218	0.018
M1_Climate	0.251	0.802	0.170	0.288	0.006
Y_Adopt	0.301	0.170	0.724	0.220	−0.080
M2_Efficacy	0.218	0.288	0.220	1.000	0.023
Ctrl_Exp	0.018	0.006	−0.080	0.023	1.000

### 4.3 Structural model assessment

Following the guidelines established by Hair et al. (2019), we conducted a comprehensive assessment of the structural model’s quality, focusing on collinearity diagnostics, explanatory power (*R*^2^), predictive relevance (*Q*^2^), and effect sizes (*f*^2^).

Initially, the examination of collinearity within the structural model revealed (see Table 5) that the Variance Inflation Factor (VIF) values for all predictor constructs ranged from 1.000 to 1.136. All VIF values remained well below the conservative threshold of 3.0, signifying a high degree of independence among predictors and the absence of severe multicollinearity.

**Table 5:** Structural model quality (*R*^2^, *Q*^2^, VIF).

Endogenous	*R* ^2^	*Q* ^2^	Max VIF
M1_Climate	0.063	0.047	1.000
M2_Efficacy	0.106	0.077	1.067
Y_Adopt	0.127	0.071	1.136

Regarding explanatory power, the results indicated that the model accounted for 12.7% of the variance in Technology Adoption (*R*^2^ = 0.127) and 10.6% in Self-Efficacy (*R*^2^ = 0.106). While these *R*^2^ values represent a weak-to-moderate level of explanation, the predictive validity of the model was further substantiated by the blindfolding procedure. Specifically, the Stone-Geisser’s *Q*^2^ values for all endogenous constructs (ranging from 0.047 to 0.077) exceeded zero, confirming that the model possesses stable predictive relevance for the key constructs.

**Figure 2:**
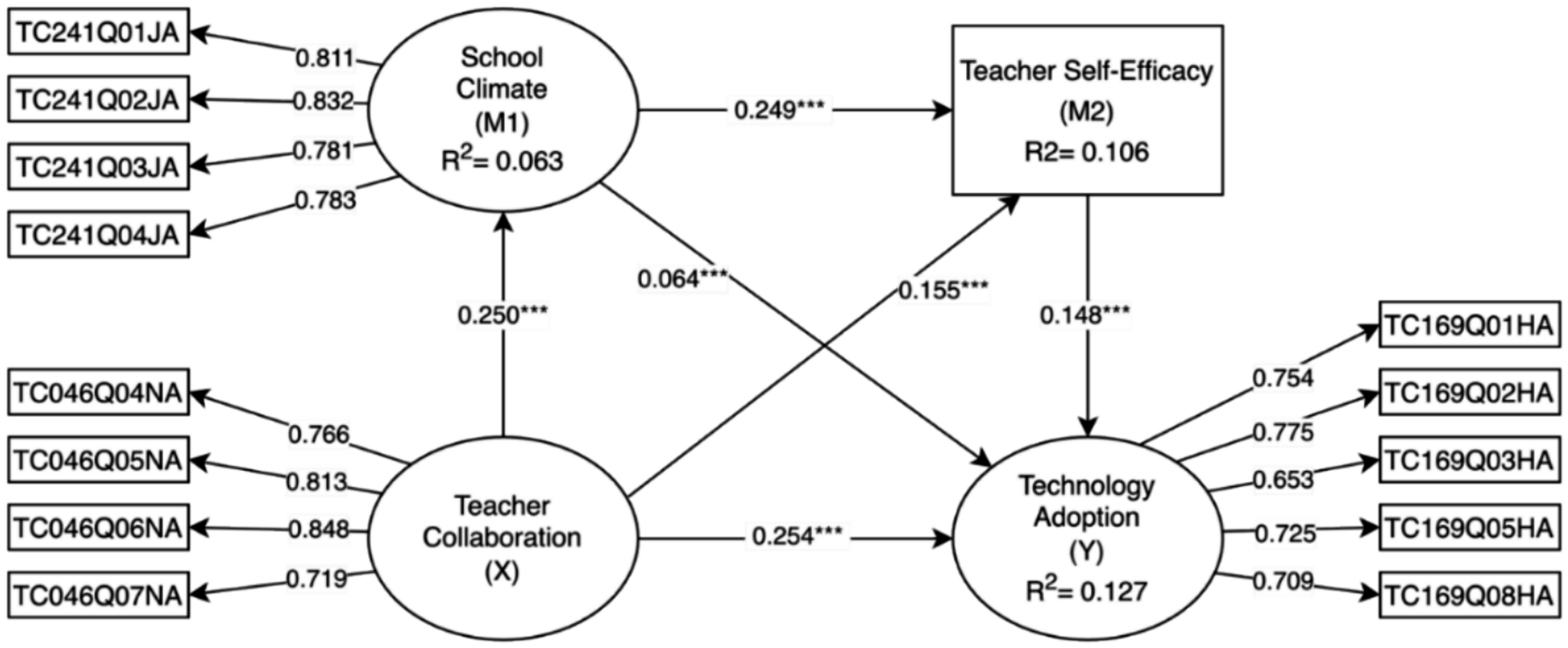
Results of the structural model analysis.

Furthermore, Table 6 delineates the substantive impact of specific paths. The results highlight that teacher collaboration played a pivotal role in driving model dynamics, with its effect sizes on technology adoption (*f*^2^ = 0.068) and school climate (*f*^2^ = 0.067) both approaching a moderate level. Notably, the effect sizes of the control variable (teaching experience) on all endogenous constructs were negligible (*f*^2^ < 0.01). This finding rules out potential confounding effects from teaching experience, thereby further bolstering the robustness of the model’s core mechanisms. The overall structural model results are depicted in Figure 2.

**Table 6:** Effect sizes (*f*^2^) for path.

Path	*f* ^2^	Effect size
X_Collab → M1_Climate	0.067	Small
Ctrl_Exp → M1_Climate	0.000	Negligible
M1_Climate → M2_Efficacy	0.065	Small
X_Collab → M2_Efficacy	0.025	Small
Ctrl_Exp → M2_Efficacy	0.000	Negligible
M2_Efficacy → Y_Adopt	0.023	Small
M1_Climate → Y_Adopt	0.004	Negligible
X_Collab → Y_Adopt	0.068	Small
Ctrl_Exp → Y_Adopt	0.009	Negligible

### 4.4 Direct path analysis

To assess the significance of the path coefficients, a bootstrapping procedure with 2,000 subsamples was conducted. The specific path coefficients (*β*), T-statistics, and significance levels are summarized in Table 7.

**Table 7:** Path coefficients and significance testing results.

Path	Beta	T-stat.	*p*-value	Sig.
X_Collab → M1_Climate	0.250	49.885	<0.001	***
Ctrl_Exp → M1_Climate	0.001	0.267	0.789	ns
M1_Climate → M2_Efficacy	0.249	49.591	<0.001	***
X_Collab → M2_Efficacy	0.155	30.117	<0.001	***
Ctrl_Exp → M2_Efficacy	0.019	3.946	<0.001	***
M2_Efficacy → Y_Adopt	0.148	30.278	<0.001	***
M1_Climate → Y_Adopt	0.064	12.396	<0.001	***
X_Collab → Y_Adopt	0.254	51.974	<0.001	***
Ctrl_Exp → Y_Adopt	−0.089	18.819	<0.001	***

Regarding the direct structural paths, the results provided support for the proposed model structure. Specifically, teacher collaboration emerged as a significant positive predictor of school climate (*β* = 0.250, *t* = 49.89, *p* < 0.001), suggesting that frequent professional interactions serve as a cornerstone for fostering a positive organizational atmosphere. Notably, collaboration also exerted a direct positive influence on teacher self-efficacy (*β* = 0.155, *t* = 30.12, *p* < 0.001) and technology adoption (*β* = 0.254, *t* = 51.97, *p* < 0.001), implying that collaborative behaviors inherently facilitate both psychological empowerment and the transfer of technical resources.

Similarly, school climate demonstrated a robust positive impact on teacher self-efficacy (*β* = 0.249, *t* = 49.59, *p* < 0.001), validating the nurturing role of a supportive environment in shaping individual beliefs. It also showed a significant, albeit smaller, direct effect on technology adoption (*β* = 0.064, *t* = 12.40, *p* < 0.001). Furthermore, teacher self-efficacy was substantiated as a vital driver, significantly promoting technology adoption (*β* = 0.148, *t* = 30.28, *p* < 0.001). These significant associations among all core variables lay the necessary structural foundation for the subsequent mediation analysis.

In terms of control variables, teaching experience showed no significant impact on school climate (*β* = 0.001, *p* > 0.05). However, it exerted marginal effects on self-efficacy (*β* = 0.019, *p* < 0.001) and technology adoption (*β* = −0.089, *p* < 0.001).

### 4.5 Mediation analysis

To delve into the intrinsic mechanisms by which teacher collaboration influences technology adoption, this study examined the mediating roles of school climate and teacher self-efficacy. Following the guidelines by Preacher and Hayes (2008), we assessed the significance of the mediation effects using bias-corrected 95% CI derived from 2,000 bootstrap subsamples. The mediation analysis results are summarized in Table 8.

**Table 8:** Mediation analysis results.

Effects/Path	Effect	95% CI	Result
Total Effect (X → Y)	0.303	[0.294, 0.312]	Significant
Direct Effect (X → Y)	0.254	[0.245, 0.264]	Significant
Total Indirect Effect	0.048	[0.045, 0.052]	Significant
Ind 1: X → M1 → Y (H1)	0.016	[0.013, 0.019]	Supported
Ind 2: X → M2 → Y (H2)	0.023	[0.021, 0.025]	Supported
Ind 3: X → M1 → M2 → Y (H3)	0.009	[0.008, 0.010]	Supported

First, the analysis of total and indirect effects revealed a complementary partial mediation pattern. As shown in Table 8, the total indirect effect of teacher collaboration on technology adoption was significant (Effect = 0.048, 95% CI [0.045, 0.052]). Since the direct effect remained robustly significant (Effect = 0.254) even after introducing the mediators, this indicates that the mediators partially explain the relationship, accounting for approximately 15.8% of the total effect. Second, the analysis of specific indirect effects provided empirical support for all three hypothesized pathways. The Environmental Pathway (H1): The indirect effect mediated solely by school climate (X → M1 → Y) was significant (Effect = 0.016, 95% CI [0.013, 0.019]). Since the 95% confidence interval did not include zero, this confirms that optimizing the organizational environment is a consistent mechanism through which collaboration promotes adoption, supporting H1. The Psychological Pathway (H2): The indirect path via teacher self-efficacy (X → M2 → Y) yielded a significant positive effect (Effect = 0.023, 95% CI [0.021, 0.025]). This finding validates that collaboration drives adoption by enhancing individual confidence, supporting H2. Notably, the size of this psychological pathway is larger than that of the environmental pathway, indicating that internal cognitive shifts are a primary driver of behavioral change. The Serial Mediation Pathway (H3): The results validated the serial mediation chain (X → M1 → M2 → Y). The specific indirect effect was significant (Effect = 0.009, 95% CI [0.008, 0.010]). This substantiates the sequential process where collaboration first builds a supportive climate, which subsequently nurtures self-efficacy and finally drives adoption behavior, fully supporting H3.

In summary, the statistical analysis portrays a clear narrative of influence: while teacher collaboration directly provides the technical resources needed for technology use, its impact is significantly amplified when it successfully builds a supportive school climate and boosts teacher confidence. The data confirms that this chain reaction—from external social interaction to environmental support, then to inner belief, and finally to behavioral action—is a statistically robust pathway for driving digital transformation.

## 5 Discussion

This study aimed to examine the mechanisms underlying the impact of teacher collaboration on technology adoption. By constructing a theoretical framework grounded in SCT and CoP, and utilizing the large-scale PISA 2022 dataset, the study empirically validated a partial mediation model. The results indicate that teacher collaboration influences technology adoption through a direct path and three distinct indirect pathways. These include the environmental optimization path through school climate (supporting H1), the psychological empowerment path through self-efficacy (supporting H2), and a sequential path where school climate enhances self-efficacy to drive adoption (supporting H3).

### 5.1 The direct impact of teacher collaboration on technology adoption

The findings reveal that the direct effect of teacher collaboration on technology adoption was the most pronounced among all structural paths. In terms of effect sizes ( *f* ^2^), teacher collaboration demonstrated a stronger predictive power ( *f* ^2^ = 0.068) compared to the direct association of school climate ( *f* ^2^ = 0.004) or self-efficacy ( *f* ^2^ = 0.023). Although these effects are classified as small-to-medium according to Cohen’s guidelines, they highlight that professional interaction is a dominant driver relative to other environmental factors in the model. This result aligns with the assertion of Datnow (2020) that interaction lies at the core of educational change, suggesting that interpersonal connectivity is a key factor in the context of digital transformation. This direct effect underscores the pragmatic nature of collaboration which, unlike administrative mandates, frequently entails concrete resource exchange and collaborative problem solving. As noted by Nguyen and Ng (2020), through activities such as co-planning and experience sharing, teachers gain direct access to the tacit knowledge of their peers, including the operational nuances of specific software and troubleshooting expertise. This knowledge spillover effect reduces the learning costs and cognitive load associated with technology use. Consequently, collaborative behaviors can translate into technology adoption via a resource exchange mechanism, bypassing the need for complex psychological transformation processes. Therefore, the presence of this direct effect explains why the mediation hypotheses (H1, H2, and H3) represent partial rather than full mediation.

### 5.2 The mediating path of school climate

This study confirmed a significant mediation pathway that originates from teacher collaboration, operates through school climate, and ultimately impacts technology adoption. This finding aligns with the social network perspective proposed by Moolenaar (2012), which posits that instructional behaviors are deeply embedded within the social fabric of the school structure. Collaboration functions as a force of social construction where high-frequency interaction dismantles the traditional egg crate structure of isolation described by Lortie (2020), thereby reshaping the organizational milieu through the establishment of norms of reciprocity. This positive climate, engendered by collaboration, serves as organizational-level social capital that provides environmental support for technology adoption. As noted by Liu et al. (2025), when teachers perceive an atmosphere of mutual trust and support, they are more inclined to experiment with digital tools within a safe environment. This willingness stems from the assurance that the school network provides a safety net if difficulties arise, rather than leaving individuals isolated. These results provide empirical support for H1.

### 5.3 The mediating path of teacher self-efficacy

This study verified a distinct independent mediation pathway proceeding from teacher collaboration, via self-efficacy, to technology adoption. This pathway illuminates the mechanism of psychological empowerment inherent in collaboration, thereby supporting the hypothesis regarding vicarious experience within SCT. As noted by Horn and Kane (2015), high-quality collaborative dialogue is pivotal for the acquisition of instructional expertise, as teachers are afforded opportunities to observe the successful practices of their peers. This modeling effect directly bolsters their conviction in their ability to command the digital classroom. On the behavioral front, high self-efficacy was proven to act as an intrinsic predictor of technology adoption. Consistent with the findings of Hatlevik (2017), this study observed that highly efficacious teachers exhibit superior agency and persistence. When confronted with the uncertainties of technology integration, such confidence predisposes teachers to view difficulties as challenges to be mastered rather than threats to be avoided, leading to more frequent and profound technology usage in instruction. Consequently, H2 is fully supported.

### 5.4 The serial mediation mechanism

The most theoretically profound finding of this study is the confirmation of the serial mediation pathway originating from collaboration, passing through school climate and self-efficacy, and culminating in technology adoption. This finding aligns with the research of Liu et al. (2025) while further unpacking the transformation from external interaction to internal psychology. This chain delineates a complete mechanism comprising socialization, internalization, and externalization. In the socialization phase, collaborative behaviors build a psychologically safe school climate through shared responsibility, echoing the views of Sin (2021) on mitigating reform tensions. Subsequently, during the internalization phase, this inclusive climate is transformed into individual teacher self-efficacy by reducing technology anxiety and providing social persuasion, a finding that aligns with the perspective of Datnow (2020) regarding agency. Finally, in the externalization phase, this psychological capital manifests as stable technology adoption behaviors. This discovery addresses the concerns of Hargreaves (1994) regarding the potential inefficacy of contrived collegiality by demonstrating that when collaboration translates into a supportive psychological climate, it can effectively drive deep-seated instructional change. Collectively, these results provide full empirical support for H3.

### 5.5 Theoretical and practical implications

The primary theoretical contribution of this study is the validation of an integrated “CoP-SCT” perspective for understanding educational change. By synthesizing CoP and SCT, this research bridges the gap between macro-level social interactions and micro-level individual cognition. Unlike prior frameworks that often treat environmental and psychological factors as isolated predictors, our findings suggest a consistent mechanism: educational reform involves a continuous process of socialization, internalization, and externalization (Vongkulluksn et al., 2018). This theoretical integration provides scholars with a scalable lens to examine how external collaboration is translated into internal psychological capital across diverse educational contexts, offering a clearer explanation for the complex dynamics of technology adoption.

Beyond this theoretical synthesis, the research offers a granular deconstruction of the multi-path mechanism through which teacher collaboration operates. Unlike prior studies that often examined isolated factors, this study distinguishes between a direct effect and three distinct indirect pathways: the environmental pathway via school climate (supporting H1), the psychological pathway via self-efficacy (supporting H2), and the serial socialization internalization pathway (supporting H3). This finding of complementary partial mediation offers a refined theoretical lens, suggesting that collaboration functions simultaneously as a pragmatic channel for resource exchange and as a transformative force for psychological empowerment.

Empirically, the study extends the boundaries of educational technology theories by validating the model within the global context of the PISA 2022 dataset. This confirmation demonstrates that the environment cognition behavior mechanism is a consistent phenomenon rather than a context-specific occurrence, thereby enhancing the generalizability of the findings across different educational systems.

Regarding practical significance, although the observed effect sizes for the mediated pathways are statistically small, they hold substantial practical value in the context of large-scale educational systems. As Kraft (2020) argues, in broad-spectrum educational interventions, even small effect sizes can translate into meaningful improvements when aggregated across thousands of schools. The identified serial mechanism suggests that small, incremental improvements in school climate can trigger a cascading positive effect on teacher efficacy and subsequent adoption, offering a cost-effective lever for policymakers.

Finally, these findings offer actionable guidance for educational practitioners at both university and school levels. For university-based teacher education, the results suggest a paradigm shift from purely technical training to the development of psychological resilience and collaborative competencies. This aligns with recent findings by Ma and Hao (2025), who highlighted that self-efficacy and behavioral adaptability are critical predictors of learning engagement in blended teaching contexts among pre-service teachers. Therefore, pre-service curricula should incorporate mandatory modules on collaborative problem-solving to cultivate future teachers’ habits of professional exchange and self-efficacy early in their careers. For K-12 school administration, the findings imply that fostering technology adoption requires institutionalizing “psychologically safe” collaborative spaces (e.g., Professional Learning Communities). By explicitly valuing peer support over top-down mandates, schools can create a nurturing climate that reduces the anxiety of digital transformation and sustains teachers’ drive for innovation.

### 5.6 Limitations and future directions

Despite its significance, this study is subject to certain limitations that warrant consideration. First, regarding the constraints of cross-sectional data, while PLS-SEM facilitates statistical inference, the cross-sectional nature of the PISA dataset precludes the establishment of strict causal relationships. Future research should employ longitudinal designs to verify the evolution of this serial mechanism over time. Second, concerning potential self-report bias, the reliance on subjective responses may make the results susceptible to common method variance. To enhance objectivity, future inquiries could triangulate these findings with student-level learning outcome data or classroom observational metrics. Third, regarding cultural context, this study utilized a global aggregate sample and did not deeply investigate the heterogeneity of the proposed mechanism across diverse cultural backgrounds. Future cross-cultural comparative studies are essential to elucidate the boundary conditions and applicability of this model under varying educational systems.

## 6 Conclusion

Guided by the research question of how to translate social interaction into behavioral change, this study utilized the PISA 2022 global dataset to examine the mediating mechanisms linking teacher collaboration to technology adoption. This study provides evidence for the complex relationship between teacher collaboration and technology adoption by validating a complementary partial mediation model. The findings suggest that teacher collaboration functions as a fundamental mechanism for both social construction and psychological empowerment. Specifically, the results confirm that collaboration facilitates technology adoption through a multi-path mechanism. Beyond its direct role in facilitating knowledge transfer, collaboration exerts indirect effects by optimizing the school climate, bolstering teacher self-efficacy, and supporting a sequential process of socialization, internalization, and externalization. These results suggest that fostering interpersonal connectivity within schools is essential for promoting deep-seated digital transformation, as it addresses both the environmental and psychological dimensions of educational change.

## Data Availability

Publicly available datasets were analyzed in this study. This data can be found here: https://www.oecd.org/en/publications/pisa-2022-results-volume-i_53f23881-en.html.

## References

[ref1] Alenezi A. (2017). Obstacles for teachers to integrate technology with instruction. Educ. Inf. Technol. 22, 1797–1816. doi: 10.1007/s10639-016-9518-5

[ref2] Bandura A. (1986). Social Foundations of Thought and Action: A Social Cognitive Theory. Englewood Cliffs, USA: Prentice-Hall, Inc.

[ref301] Bandura A. (1997). Self-efficacy: The exercise of control. New York, NY: W. H. Freeman. Available at: https://psycnet.apa.org/record/1997-08589-000

[ref3] Bandura A. (2001). Social cognitive theory: an agentic perspective. Annu. Rev. Psychol. 52, 1–26. doi: 10.1146/annurev.psych.52.1.1 11148297

[ref4] Chin W. W. (1998). “The partial least squares approach to structural equation modeling,” in Modern Methods for Business Research. ed. Marcoulides, G. (Mahwah, USA: Psychology Press), 295–336.

[ref5] Çoban Ö. Özdemir N. Bellibaş M. Ş. (2023). Trust in principals, leaders’ focus on instruction, teacher collaboration, and teacher self-efficacy: testing a multilevel mediation model. Educ. Manage. Adm. Leadersh. 51, 95–115. doi: 10.1177/1741143220968170

[ref6] Cohen J. McCabe E. M. Michelli N. M. Pickeral T. (2009). School climate: research, policy, practice, and teacher education. Teach. Coll. Rec. 111, 180–213. doi: 10.1177/016146810911100108

[ref7] Compeau D. R. Higgins C. A. (1995). Computer self-efficacy: development of a measure and initial test. MIS Q. 19, 189–211. doi: 10.2307/249688

[ref8] Datnow A. (2020). The role of teachers in educational reform: a 20-year perspective. J. Educ. Change 21, 431–441. doi: 10.1007/s10833-020-09372-5

[ref9] Dong Y. Xu C. Chai C. S. Zhai X. (2020). Exploring the structural relationship among teachers’ technostress, technological pedagogical content knowledge (TPACK), computer self-efficacy and school support. Asia Pac. Educ. Res. 29, 147–157. doi: 10.1007/s40299-019-00461-5

[ref10] Egodawatte G. McDougall D. Stoilescu D. (2011). The effects of teacher collaboration in grade 9 applied mathematics. Educ. Res. Policy Pract. 10, 189–209. doi: 10.1007/s10671-011-9104-y

[ref11] Ertmer P. A. Ottenbreit-Leftwich A. T. Sadik O. Sendurur E. Sendurur P. (2012). Teacher beliefs and technology integration practices: a critical relationship. Comput. Educ. 59, 423–435. doi: 10.1016/j.compedu.2012.02.001

[ref12] Fornell C. Larcker D. F. (1981). Evaluating structural equation models with unobservable variables and measurement error. J. Mark. Res. 18, 39–50. doi: 10.1177/002224378101800104

[ref13] Fraillon J. (2025). “Introduction to the IEA international computer and information literacy study 2023,” in ed. Fraillon J An International Perspective on Digital Literacy: Results from ICILS 2023. (Amsterdam, Netherlands: Springer), 1–17.

[ref14] Friesen S. Brown B. (2022). Teacher leaders: developing collective responsibility through design-based professional learning. Teach. Educ. 33, 254–271. doi: 10.1080/10476210.2020.1856805

[ref15] Gordon D. Blundell C. Mills R. Bourke T. (2023). Teacher self-efficacy and reform: a systematic literature review. Aust. Educ. Res. 50, 801–821. doi: 10.1007/s13384-022-00526-3

[ref16] Hair J. F. Hult G. T. M. Ringle C. M. Sarstedt M. Danks N. P. Ray S. (2021). Partial least Squares Structural Equation Modeling (PLS-SEM) Using R: A Workbook. Cham, Switzerland: Springer Nature.

[ref17] Hair J. F. Risher J. J. Sarstedt M. Ringle C. M. (2019). When to use and how to report the results of PLS-SEM. Eur. Bus. Rev. 31, 2–24. doi: 10.1108/EBR-11-2018-0203

[ref18] Haleem A. Javaid M. Qadri M. A. Suman R. (2022). Understanding the role of digital technologies in education: a review. Sustain. Operat. Comput. 3, 275–285. doi: 10.1016/j.susoc.2022.05.004

[ref19] Hargreaves A. (1994). Changing Teachers, Changing times: Teachers’ work and Culture in the Postmodern age. London and New York, NY: Continuum & Teacher’s College Press.

[ref20] Hargreaves A. (2021). Teacher collaboration: 30 years of research on its nature, forms, limitations and effects. Policy Teach. Educ. Qual. Teach. Teach. ed. Christopher Day. New York, USA. 103–121.

[ref21] Hatlevik O. E. (2017). Examining the relationship between teachers’ self-efficacy, their digital competence, strategies to evaluate information, and use of ICT at school. Scand. J. Educ. Res. 61, 555–567. doi: 10.1080/00313831.2016.1172501

[ref22] Henderson J. Corry M. (2021). Teacher anxiety and technology change: a review of the literature. Technol. Pedagogy Educ. 30, 573–587. doi: 10.1080/1475939x.2021.1931426

[ref23] Horn I. S. Kane B. D. (2015). Opportunities for professional learning in mathematics teacher workgroup conversations: relationships to instructional expertise. J. Learn. Sci. 24, 373–418. doi: 10.1080/10508406.2015.1034865

[ref24] Howard S. K. (2013). Risk-aversion: understanding teachers’ resistance to technology integration. Technol. Pedagogy Educ. 22, 357–372. doi: 10.1080/1475939x.2013.802995

[ref25] Howard S. K. Gigliotti A. (2016). Having a go: looking at teachers’ experience of risk-taking in technology integration. Educ. Inf. Technol. 21, 1351–1366. doi: 10.1007/s10639-015-9386-4

[ref26] Hoy W. K. Sweetland S. R. (2001). Designing better schools: the meaning and measure of enabling school structures. Educ. Adm. Q. 37, 296–321. doi: 10.1177/00131610121969334

[ref27] Hülshoff A. Nonte S. Reintjes C. (2025). Pre-service teachers’ uncertainty tolerance regarding the in-classroom use of information and communications technology: predictors, concerns and perceived training needs. J. Educ. Teach. 51, 541–565. doi: 10.1080/02607476.2025.2502825

[ref28] Karasavvidis I. Kollias V. (2017). “Understanding technology integration failures in education: the need for zero-order barriers,” in eds. Sidorkin, A. M. and Warford M. K. Reforms and Innovation in Education: Implications for the Quality of human Capital. (Cham, Switzerland: Springer), 99–126.

[ref29] Khlaif Z. N. Sanmugam M. Joma A. I. Odeh A. Barham K. (2023). Factors influencing teacher’s technostress experienced in using emerging technology: a qualitative study. Technol. Knowl. Learn. 28, 865–899. doi: 10.1007/s10758-022-09607-9

[ref30] Kraft M. A. (2020). Interpreting effect sizes of education interventions. Educ. Res. 49, 241–253. doi: 10.3102/0013189x20912798

[ref31] Lampert M. (2010). Learning teaching in, from, and for practice: what do we mean? J. Teach. Educ. 61, 21–34. doi: 10.1177/0022487109347321

[ref32] Lave J. Wenger E. (1991). Situated Learning: Legitimate Peripheral Participation. Cambridge, England: Cambridge University Press.

[ref33] Li S. (2023). The effect of teacher self-efficacy, teacher resilience, and emotion regulation on teacher burnout: a mediation model. Front. Psychol. 14:1185079. doi: 10.3389/fpsyg.2023.1185079, 37691805 PMC10483148

[ref34] Li Y. (2024). Characterising student teachers’ noticing habits in technology-enhanced dialogic reflection. Educ. Sci. 14:1393. doi: 10.3390/educsci14121393

[ref35] Little J. W. (1990). The persistence of privacy: autonomy and initiative in teachers' professional relations. Teach. Coll. Rec. 91, 509–536. doi: 10.1177/016146819009100403

[ref36] Liu J. Darko E. N. K. O. Aziku M. (2025). Relationship between digital preparedness and digital integration: mediation evidence on the role of school climate. J. New Approaches Educ. Res. 14:18. doi: 10.1007/s44322-025-00040-1

[ref37] Lortie D. C. (2020). Schoolteacher: A Sociological Study. Chicago, USA: University of Chicago Press.

[ref38] Ma K. Hao T. (2025). Predictors of pre-service teachers’ learning engagement in blended teaching. Distance Educ. 46, 477–495. doi: 10.1080/01587919.2024.2387035

[ref39] Manouchehri A. (1997). School mathematics reform: implications for mathematics teacher preparation. J. Teach. Educ. 48, 197–209. doi: 10.1177/0022487197048003005

[ref40] Maral M. (2025). Sixty years of school climate research: a bibliometric and content analysis. Psychol. Sch., 1–32. doi: 10.1002/pits.70129

[ref41] Mather B. R. Visone J. D. (2024). Peer observation to improve teacher self-efficacy. J. Educ. Res. Pract. 14, 1–22. doi: 10.5590/jerap.2024.14.01

[ref42] Min M. (2023). School culture, self-efficacy, outcome expectation, and teacher agency toward reform with curricular autonomy in South Korea: a social cognitive approach. Asia Pac. J. Educ. 43, 951–967. doi: 10.1080/02188791.2019.1626218

[ref43] Moolenaar N. M. (2012). A social network perspective on teacher collaboration in schools: theory, methodology, and applications. Am. J. Educ. 119, 7–39. doi: 10.1086/667715

[ref44] Nguyen D. Ng D. (2020). Teacher collaboration for change: sharing, improving, and spreading. Prof. Dev. Educ. 46, 638–651. doi: 10.1080/19415257.2020.1787206

[ref45] Ninković S. Florić O. K. Đorđić D. (2022). The effect of teacher trust in colleagues on collective teacher efficacy: examining the mediating role of the characteristics of professional learning communities. Teach. Teach. Educ. 119:103877. doi: 10.1016/j.tate.2022.103877

[ref46] Nordlöf C. Hallström J. Höst G. E. (2019). Self-efficacy or context dependency?: exploring teachers’ perceptions of and attitudes towards technology education. Int. J. Technol. Des. Educ. 29, 123–141. doi: 10.1007/s10798-017-9431-2

[ref47] OECD (2023). PISA 2022 Results (volume II): learning During–and From–Disruption. Paris, France: OECD Publishing.

[ref48] Ostovar-Nameghi S. A. Sheikhahmadi M. (2016). From teacher isolation to teacher collaboration: theoretical perspectives and empirical findings. Engl. Lang. Teach. 9, 197–205. doi: 10.5539/elt.v9n5p197

[ref49] Preacher K. J. Hayes A. F. (2008). Asymptotic and resampling strategies for assessing and comparing indirect effects in multiple mediator models. Behav. Res. Methods 40, 879–891. doi: 10.3758/BRM.40.3.879 18697684

[ref50] Reilly K. A. (2017). Observing peers develops practice, changes culture. Phi Delta Kappan 98, 13–18. doi: 10.1177/0031721717696472

[ref51] Ryan J. Kang C. Mitchell I. Erickson G. (2009). China's basic education reform: an account of an international collaborative research and development project. Asia Pac. J. Educ. 29, 427–441. doi: 10.1080/02188790903308902

[ref52] Sin K. K. T. (2021). School-university partnerships in teacher education: tension between partners and how they handle it. GiLE J. Skills Dev. 1, 87–98. doi: 10.52398/gjsd.2021.v1.i1.pp87-98

[ref53] Sun T. Yoon M. (2025). The impact of digital transformation on faculty performance in higher education: the mediating role of digital self-efficacy and the moderating role of task-technology fit. Front. Psychol. 16:1693375. doi: 10.3389/fpsyg.2025.1693375, 41190117 PMC12580117

[ref54] Tabancalı E. (2016). The relationship between teachers’ job satisfaction and loneliness at the workplace. Eurasian J. Educ. Res. 16, 263–280.

[ref55] Tatar C. Jiang S. Rosé C. P. Chao J. (2025). Exploring teachers’ views and confidence in the integration of an artificial intelligence curriculum into their classrooms: a case study of curricular co-design program. Int. J. Artif. Intell. Educ. 35, 702–735. doi: 10.1007/s40593-024-00404-2

[ref56] Tirri K. (2014). The last 40 years in Finnish teacher education. J. Educ. Teach. 40, 600–609. doi: 10.1080/02607476.2014.956545

[ref57] Tripon C. (2022). Supporting future teachers to promote computational thinking skills in teaching stem—a case study. Sustainability 14:12663. doi: 10.3390/su141912663

[ref58] Vangrieken K. Dochy F. Raes E. Kyndt E. (2015). Teacher collaboration: a systematic review. Educ. Res. Rev. 15, 17–40. doi: 10.1016/j.edurev.2015.04.002

[ref59] Venkatesh V. Morris M. G. Davis G. B. Davis F. D. (2003). User acceptance of information technology: toward a unified view. MIS Q. 27, 425–478. doi: 10.2307/30036540

[ref60] Vongkulluksn V. W. Xie K. Bowman M. A. (2018). The role of value on teachers' internalization of external barriers and externalization of personal beliefs for classroom technology integration. Comput. Educ. 118, 70–81. doi: 10.1016/j.compedu.2017.11.009

[ref61] Weddle H. (2022). Approaches to studying teacher collaboration for instructional improvement: a review of literature. Educ. Res. Rev. 35:100415. doi: 10.1016/j.edurev.2021.100415

[ref62] Wenger E. (1998). Communities of practice: learning as a social system. Syst. Thinker 9, 2–3. doi: 10.1017/CBO9780511803932

[ref63] Yan L. Lin Y. Li W. Hu C. (2025). Exploring the interplay of mindfulness, self-efficacy, and burnout among Chinese preschool teachers: a network approach. Front. Psychol. 16:1483099. doi: 10.3389/fpsyg.2025.1483099, 40144030 PMC11937131

